# Step count recovery patterns in the first six weeks after knee replacement in individuals with knee osteoarthritis: a secondary analysis of a prospective observational cohort study using wrist-worn accelerometry

**DOI:** 10.1007/s00296-026-06135-y

**Published:** 2026-06-04

**Authors:** Ayobami E. Olanrewaju, Emma Pritchard, Shuai Shao, Andrew J. Price, Aiden Doherty, Sabine N. van der Veer, David C. Wong, Scott R. Small, Stephanie R. Filbay, William G. Dixon

**Affiliations:** 1 School of Health Sciences, Division of Informatics, Imaging and Data Sciences, University of Manchester, Manchester, M13 9PT UK; 2 Centre for Health, Exercise and Sports Medicine, Department of Physiotherapy, University of Melbourne, Parkville, Melbourne, VIC 3000 Australia; 3 Nuffield Department of Orthopaedics, Rheumatology and Musculoskeletal Sciences, University of Oxford, Oxford, UK; 4 Nuffield Department of Population Health, University of Oxford, Oxford, UK; 5 University of Leeds, Leeds Institute of Health Sciences, Leeds, UK; 6 NIHR Manchester Biomedical Research Centre, Manchester University NHS Foundation Trust, Manchester Academic Health Science Centre, Manchester, UK

**Keywords:** Knee osteoarthritis, Knee arthroplasty, Physical activity, Postoperative period

## Abstract

Effective patient education and activity expectation-setting following knee replacement requires understanding postoperative recovery patterns. We investigated step-count recovery patterns, identified recovery trajectory clusters, and examined differences in postoperative outcomes and preoperative factors associated with those clusters. Using wrist-worn accelerometer, step-counts were measured for one week preoperatively and six weeks postoperatively in patients with knee osteoarthritis undergoing total or unicompartmental knee replacement (UKR). Patient-reported outcomes (OKS, EQ-5D-3L, EQ-VAS) were collected preoperatively and at six postoperative weeks. The proportion of participants exceeding their preoperative step-count was calculated. Latent class growth analysis identified trajectories of absolute (postoperative) and relative (postoperative-to-preoperative ratio) step-counts. Kruskal–Wallis with Dunn’s test assessed differences in postoperative outcomes across trajectories, and multinomial logistic regression assessed associations between trajectories and preoperative factors. Eighty-two participants (aged 42–89 years; 52% women) were included. Within six weeks, 32% exceeded preoperative step-counts. Three trajectories (high, moderate and low recovery) were identified for both absolute and relative step-counts. For *absolute* steps, the “high recovery” group (*n* = 30, 37%) began with a median of 991 steps [IQR 1,335] at Week1 and achieved a median of 6,606 steps [IQR 2,940] by Week6. This compares to the “moderate” group (*n* = 24, 29%) who improved from 360 steps [IQR 481] at Week1 to 3,739 steps [IQR 1,918] at Week6, and the “low” group (*n* = 28, 34%) who increased from 29 steps [IQR 176] at Week1 to 1,452 steps [IQR 1,504] by Week6. For *relative* steps, the “high recovery” group (*n* = 39, 47%) reached 92% [IQR 29%] of their preoperative step-count at Week6, compared with 64% [IQR 25%] in the “moderate” group (*n* = 26, 32%) and 31% [IQR 7%] in the “low” group (*n* = 17, 21%). Patients in the high recovery trajectories reported less pain, better knee function (OKS) and better health status at six weeks versus low recovery trajectories. Membership of the high trajectories was more likely in those with higher preoperative step-count and for those managed with UKR. Patients with lower preoperative step-count were unlikely to achieve high absolute step-count and less likely to achieve high- or moderate-relative recovery trajectories versus those with a higher preoperative step-count. One in three individuals regained preoperative step-count by six weeks, with heterogeneity in recovery patterns. Greater recovery was associated with higher preoperative step-count and UKR procedures.

**Supplementary Information:**

The online version contains supplementary material available at 10.1007/s00296-026-06135-y.

## Introduction

Knee osteoarthritis (OA) is the most common form of OA [1, 2] with many individuals requiring knee replacement surgery to relieve pain and restore function. In the UK, over 120,000 total knee replacement (TKR) or unicompartmental knee replacement (UKR) are performed yearly [3, 4], the majority for knee OA [4]. Informed decision-making about surgery depends on patients and clinicians understanding likely outcomes [5], including changes in pain, mobility, and quality of life (QoL) in the long-term, as well as anticipated recovery patterns in the weeks and months following surgery. Establishing realistic expectations is essential as it helps patients prepare for life after surgery and reduces dissatisfaction with surgical outcomes [6, 7].

Recovery following knee replacement occurs over several months, with the early postoperative phase differing markedly from later stages, as shown in studies of pain and patient-reported outcomes [8–10]. During the first six weeks (early phase), the incision heals [11], patients transition from walking aids to independent mobility, and pain subsides [11, 12]. Beyond this time, pain continues to improve [13–15] and most patients resume normal activities such as work [16, 17] and physical activity (PA) [18] with ongoing improvement in QoL [15]. Recovery trajectories for pain and QoL have been described in the short [10] and long-term [8, 9, 19], with early six-week trajectories shown to predict one year postoperative outcomes [10].

While pain and QoL trajectories have been explored, objective quantification of PA recovery trajectories within the first six weeks remains limited. Time-varying patterns of activity recovery are important to know if, prior to surgery, patients are to understand what to expect postoperatively, and consequently whether their actual activity recovery is better or worse than expected. Understanding patterns of activity recovery might also enable early identification of poor progress for clinical teams, allowing targeted interventions such as more intensive physiotherapy.

Traditional self-reported questionnaires about PA are known to be imprecise with recall error [20] and burdensome if needed frequently. Wearable accelerometers [21] offer a promising means of passively monitoring PA recovery, minimising recall error [20] and reducing burden. PA metrics like step-count are understandable to patients, given their widespread use in wearable and consumer accelerometer-based devices such as smartphones and fitness trackers. Several studies have previously reported on early postoperative step-count recovery. Some, however, considered only discrete measurement time points (e.g., preoperative and six-week assessments) [22–25]. Other studies that did report daily measures throughout early recovery reported an average result across the population [26–32], which prevents an understanding of whether there are patterns of ‘good’ and ‘poor’ responders and what might differentiate those people, while others have started to consider whether distinct recovery patterns can be identified [33, 34]. Knowing about different recovery patterns can inform clinical decisions, which in turn can help optimise patient outcomes.

This study aims to (1) investigate step-count recovery patterns, (2) identify clusters of step-count recovery trajectories, (3) examine the association between postoperative outcomes and recovery trajectory clusters, and (4) identify preoperative factors associated with recovery trajectory clusters. We hypothesised that recovery patterns would be heterogeneous, associated with postoperative outcome measures, and that preoperative factors would be associated with recovery trajectory clusters.

## Methods

### Design

This is a secondary analysis of a single-site prospective study [35] that investigated compliance with the use of accelerometery for PA monitoring and examined correlations between accelerometry-derived PA measures and patient-reported outcome measures in people who underwent knee replacement for OA. This study follows the Strengthening the Reporting of Observational Studies in Epidemiology (STROBE) guidelines. The completed STROBE checklist is provided in Supplementary File-STROBE-Checklist.

### Participants

In the parent study, patients scheduled for UKR or TKR at the Nuffield Orthopaedic Centre (Oxford University Hospitals, National Health Service (NHS) Trust, Oxford, UK) between February 2020 and July 2021 were identified through the outpatient assessment list [35]. Participants were eligible if they were: 18 years or older, able and willing to provide informed consent, and undergoing knee replacement for the treatment of OA. They were excluded if they had a disease or illness that could affect their participation or were enrolled in another study that might influence their step-count. Following the standard care pathway employed at this centre, patients underwent UKR if their OA was isolated to one tibio-femoral compartment of the knee with an intact anterior cruciate ligament. When these conditions were not met, patients underwent TKR. All patients underwent routine postoperative rehabilitation under the direction of physiotherapists. Inclusion in the study did not change patients’ normal NHS preoperative or postoperative clinical care pathway.

Approximately two weeks before their preoperative assessment, potential participants were sent an invitation letter. At the assessment, researchers confirmed eligibility, addressed questions, and enrolled participants. Those who agreed to participate provided written informed consent, approved by the South West-Frenchay Research Ethics Committee (reference: 19/SW/0151). For the present analysis, participants were included if they had preoperative step-count data and at least one day of step-count data in each week from week two to week five postoperatively, ensuring that their recovery trajectory could be reliably estimated.

### Measures

Step-count: Participants were provided with an Axivity AX3 activity tracker (Axivity, Newcastle, UK), a research-grade triaxial accelerometer with a 25 Hz sampling rate and a dynamic range of ±8 g. The device collected raw accelerometry data, which were extracted using the OMGui software package (OpenMovement, Newcastle, UK, v.1.0.0.43) and processed into step-count. Details of the step-count derivation are provided in Supplementary File-1. Participants were instructed to wear the device on their dominant wrist continuously (24 h per day) for seven consecutive days within a period no more than three months before surgery, and again for six weeks after surgery. Each participant was provided with the device and returned it to the researchers by post. Device-measured step-count was chosen as the primary PA metric in the present analysis to ensure comparability with previous research and because it provides a practical estimate of walking [36], which patients often identify as a key recovery goal after TKR [7].

Other measures: Demographic data, including sex, age, and body mass index (BMI), were collected at study enrolment. Routinely administered patient-reported outcome measures were obtained preoperatively and at six weeks postoperatively. These included the Oxford Knee Score (OKS), the EuroQol 5-Dimension 3-Level (EQ-5D-3L) index score, and the EuroQol Visual Analogue Scale (EQ-VAS).

The OKS is a 12-item, patient-administered questionnaire designed to assess pain and disease severity following knee replacement, with scores ranging from 0 (most severe symptoms) to 48 (least severe symptoms) [37]. It has good validity (correlations of 0.4–0.8 with the Knee Injury and Osteoarthritis Outcome Score questionnaire) [38], reliability (Cronbach’s alpha > 0.85 [38]; internal consistency (≥ 0.7) [39]), and responsiveness before and after knee replacement.

The EQ-5D-3L index summarises health status on a scale from − 0.594 to 1, where values below 0 indicate health states considered worse than death and 1 indicates full health [40]. It has shown good reliability (Cronbach’s alpha > 0.70) and responsiveness before and after a knee replacement [41]. The EQ-5D comprises two components: the EQ-5D-3L descriptive system, which generates the index score, and the EQ-VAS, which measures overall health-related quality of life on 0-100 scale (0 = worst imaginable health; 100 = best imaginable health). The EQ-VAS has demonstrated predictive validity comparable to the OKS [42] in populations with knee replacements.

Postoperatively, participants also completed a daily paper-based pain diary, responding to the question: ‘Using the pain scale provided (0–10), how would you rate your average knee pain over the past 24 hours?’ where 0 indicated no pain and 10 the worst possible pain. Additional data, including type of knee replacement (UKR or TKR) and six-week postoperative knee flexion range of motion measured by a physiotherapist, were extracted from medical records.

### Statistical analysis

All data analyses were conducted in R version 4.4.3.

Baseline characteristics of all participants were summarised using medians and interquartile ranges for continuous variables and counts and percentages for categorical variables.

#### Step-count recovery patterns

Preoperative step-count was available as a median value of seven days of data collected within three months before surgery. Step-count data for the first two postoperative days was not available for all participants due to delays in receiving their devices in the post and were thus excluded from the analysis. Postoperative step-count was therefore available from Day3 to Day42.

Patterns of recovery over time were examined by plotting absolute postoperative daily step-counts across the six-week period. Recovery was also evaluated in terms of the proportion of preoperative activity regained, expressed as the ratio of postoperative to preoperative step-counts (postoperative/preoperative step-count) and plotted separately. Given that preoperative step-count was represented by each participant’s median preoperative step-count, a centred seven-day rolling median (ignoring missing values) was applied to the postoperative data to obtain comparable values on the same scale. The centred median was chosen because it provides an estimate anchored to the day of interest. The ratio of these rolling averages (postoperative/preoperative step-count) was calculated for every participant across the follow-up period as the relative step-count. The cumulative proportion of participants who achieved their preoperative activity level through time, based on the daily rolling averages, was calculated and presented graphically.

#### Identify clusters of step-count recovery trajectories

Latent class growth analysis (LCGA) was applied to postoperative step-counts (absolute and relative values) using the lcmm package [43, 44] in R. LCGA identifies unobserved subgroups that share similar patterns of change over time [45], and assumes no within-class variance. This assumption implies that individuals assigned to the same class follow identical trajectories. Given that the aim of the analysis is to identify broad, clinically interpretable patterns of post-operative recovery rather than to model individual-level variability, LCGA was selected as an appropriate method. It assigns members to latent groups based on posterior group membership probabilities (i.e., the likelihood that a member belongs to a given group). Members of the same group have a higher probability of belonging to that group and following its corresponding trajectory than to any other group or trajectory. To assess the impact of the assumption of no within-class variance, models with different numbers of classes (groups) were examined in terms of class size, and posterior group membership to determine how well individuals fit the assigned trajectory groups.

Models with one to five groups were fitted for absolute and relative step-count. A quadratic function of days since surgery (fixed effect) was included in the model to describe the average trajectory of steps over time, while repeated measures within individuals were accounted for by specifying the individual identifier as the clustering variable to ensure that the model captured within-person dependence. A nonlinear spline-based link function was included to allow the outcome scale to accommodate non-normal, skewed or extreme values in the step data. Model performance was evaluated using the Bayesian Information Criterion (BIC), entropy, posterior group membership probability, and the number and percentage of participants per group. The optimal model was selected based on these criteria, requiring at least 10% of participants in each group, a posterior group probability of ≥ 0.7 for each trajectory to ensure reliable group separation and demonstrating clinical face validity to the research team to ensure that the identified trajectories were interpretable, meaningful, and reasonably distinct.

The mean trajectory with its 95% confidence interval for each identified trajectory group was plotted separately for absolute and relative step-count.

#### Examine the association between postoperative outcomes and recovery trajectory clusters

After identifying the trajectories, postoperative outcomes obtained at six weeks (EQ-5D-3L index, OKS, EQ-VAS, and postoperative knee flexion range of motion), as well as median and maximum pain scores computed for week one and week six, were summarised by trajectory group using medians and interquartile ranges. Between-trajectory differences were assessed using the Kruskal–Wallis test. Dunn’s test with Holm’s correction was applied to factors showing significant differences (*p* < 0.05) to identify which specific trajectory clusters differed. The Holm’s correction adjust p-values and control the familywise error rate from multiple testing, thereby reducing the risk of Type I errors (false positives) [46].

#### Identify preoperative factors associated with the recovery trajectory clusters

Baseline characteristics associated with trajectory membership were examined using multinomial logistic regression at a 5% significance level. Preoperative factors (EQ-5D-3L index, OKS, and EQ-VAS) were assessed for multicollinearity using correlation analysis, and variance inflation factors.

To investigate whether preoperative step-count influences recovery trajectories, participants’ median preoperative step-counts were divided into tertiles (high, medium and low). A Sankey diagram was used to visualise how participants transitioned from tertiles of preoperative step-count to the postoperative trajectories for absolute and relative step-count.

## Results

### Participant characteristics

Eighty-two of the 97 participants from the parent study met eligibility requirements for the current study and were included in all analyses. Their baseline characteristics, stratified by knee replacement type, are presented in Table 1. The cohort comprised individuals aged 42–89 years, 52% were women, 37% were overweight and 48% were obese. Included participants provided a median of 39 days of step-count data (range 12–40 days), representing an overall completeness of 93% (3047 out of 3280 [82 individuals x 40 days]) in the postoperative period (Supplementary File-2). The median preoperative and postoperative daily device wear time were 24 h (postoperative range: 15–24 h). Of the fifteen people in the parent study who did not meet the eligibility criteria, one lacked preoperative step-count data, and 14 did not have at least one day of data in each week from Week2 to Week5. Compared with included participants, those excluded were more likely to be older (46.7% aged 72–89 years versus 38%), obese (53.3% versus 48%), and to have undergone UKR (60% versus 55%).

**Table 1:** Baseline characteristics of the study participants

Characteristics	All participants (*N* = 82)	Knee replacement surgery	
		Unicompartmental (*N* = 45)	Total (*N* = 37)
Sex, N (%)			
Female	43 (52)	24 (53)	19 (51)
Male	39 (48)	21 (47)	18 (49)
Age, years, N (%)			
42–60	18 (22)	11 (24)	7 (19)
> 60–72	33 (40)	17 (38)	16 (43)
> 72–89	31 (38)	17 (38)	14 (38)
BMI, kg/m^2^, N (%)			
Normal weight	13 (16)	7 (16)	6 (16)
Overweight	30 (37)	19 (42)	11 (30)
Obesity Class I to III	39 (48)	19 (42)	20 (54)

*BMI* body mass index, *N* number

#### Step-count recovery patterns

**Fig. 1:**
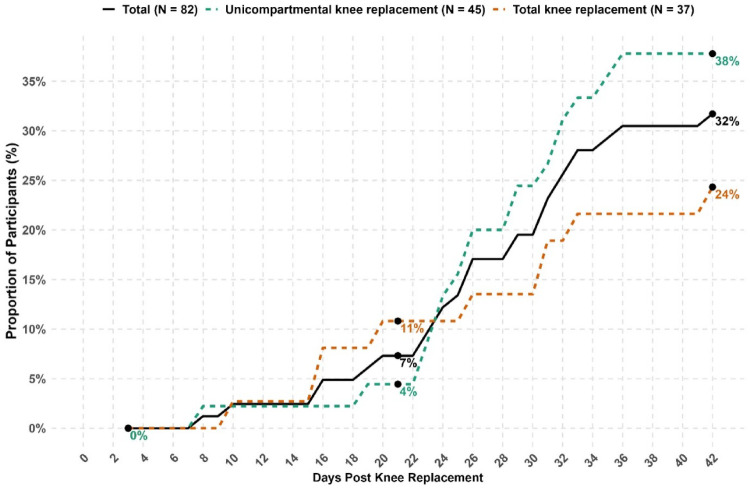
Proportion of participants exceeding their preoperative step-count at least once during the six weeks postoperative period

**Fig. 2:**
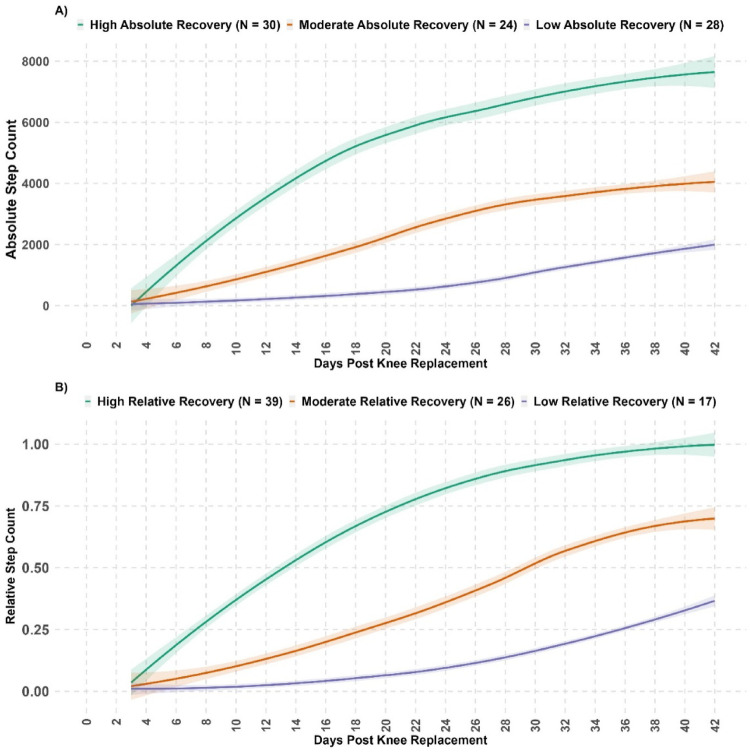
Three trajectories of step-count recovery within six weeks after knee replacement for absolute step-count (upper panel: **A**) and relative step-count (lower panel: **B**)

Participants’ step-count typically dropped markedly in the immediate postoperative period compared to their preoperative baseline, then gradually increased to different extents and at different rates (Supplementary File-3). By the end of six weeks, 32% of participants had achieved their preoperative step-count at least once (38% UKR and 24% TKR) (Fig. 1). A greater proportion of younger participants (≤ 72 years) reached their preoperative step-count compared with those in the oldest age group (> 72 years), with no differences between males and females (Supplementary File-4 Figs. 1 and 2).

#### Clusters of step-count recovery trajectories

We identified three recovery trajectories over the six weeks following knee replacement surgery (Fig. 2). The three-trajectory model was selected based on lower BIC, high entropy indicating clear class separation, high posterior group membership probability (> 0.90) for each trajectory, and because it was considered clinically meaningful by the research team compared with alternative solutions (Supplementary File-5).

All trajectories had increasing step-counts from postoperative Day3, although the rate and magnitude of recovery varied. Based on absolute postoperative step-counts (Fig. 2A), the high-recovery trajectory, which included the largest proportion of participants (37%), demonstrated a rapid, steady increase until Day20 (approximately three weeks), after which gains continued at a slower rate, rising from a median of 991 [interquartile range (IQR) 1335] steps at Week1 to 6606 [IQR 2940] steps at Week6. The moderate-recovery trajectory (29%) also showed consistent improvement, though to a lesser extent than the high-recovery trajectory; gains were sustained until around Day32 (approximately five weeks) after which progress became slower, with steps rising from 360 [IQR 481] at Week1 to 3,739 [IQR 1918] steps at Week6. The low-recovery trajectory (34%) exhibited minimal early gains, with improvement observed after three weeks but achieving only modest increases by Week6, rising from a median of 29 steps [IQR 176] to 1452 [IQR 1504] steps.

When recovery trajectories were examined relative to preoperative step-counts rather than absolute values (Fig. 2B), the high-recovery group reached a median value of 92% [IQR 29%] of their preoperative step-count at Week6. The moderate-recovery group reached a median value of 64% [IQR 25%] of their preoperative level, whereas the low-recovery group achieved a median value of 31% [IQR 7%] at Week6.

#### Association between postoperative outcomes and recovery trajectory clusters

With reference to absolute step-counts, participants in the high-absolute recovery group reported less pain, better OKS, and better health status than those in the low-absolute recovery group (Table 2). When recovery trajectories were defined using relative step-counts, the moderate-relative recovery group also reported less pain, better OKS, and better health status than the low-relative recovery group (Supplementary File-6).

**Table 2:** Postoperative patient-reported outcomes by absolute postoperative step-count recovery trajectory

Postoperative outcomes	High absolute recovery (*N* = 30)	Moderate absolute recovery (*N* = 24)	Low absolute recovery (*N* = 28)	^*^Kruskal Wallis		Dunn’s test			
				KW $\:{\chi\:}^{2}$	*P* value		High absolute recovery vs. moderate (*P* value)	High absolute recovery vs. low (*P* value)	Moderate absolute recovery vs. low (*P* value)
Maximum pain (week one)	7.0 (3.0)	8.0 (2.8)	8.0 (2.8)	2.09	0.351		–	–	–
Median pain (week one)	4.8 (2.8)	6.5 (4.1)	7.0 (2.4)	7.69	0.021		0.352	0.017	0.176
Maximum pain (week six)	3.0 (2.0)	2.8 (2.9)	4.0 (1.0)	4.74	0.094		–	–	–
Median pain (week six)	2.0 (2.0)	2.0 (2.4)	3.0 (1.8)	3.92	0.141		–	–	–
Oxford knee score	34.0 (11.0)	32.0 (11.0)	25.0 (9.3)	14.41	< 0.001		0.135	< 0.001	0.095
EQ_VAS	80.0 (20.0)	83.0 (17.3)	72.5 (20.8)	4.33	0.115		–	–	–
EQ_5D_3L index	0.7 (0.3)	0.7 (0.1)	0.6 (0.2)	10.91	0.004		0.347	0.004	0.072
Knee flexion	75.0 (10.0)	75.0 (27.5)	75.0 (33.0)	0.52	0.772		–	–	–

All outcomes were reported as median (interquartile range). KW = Kruskal–Wallis test, a nonparametric test for group differences. Dunn’s test = post-hoc test used to determine where differences exist between the identified trajectories. *Degree of Freedom for the Kruskal Wallis test = 2. χ^2^ = chi-square. High = High recovery trajectory; Moderate = moderate recovery trajectory; Low = Low recovery trajectory; EQ_5D_3L index = EuroQol 5-Dimension 3-Level United Kingdom index score (a measure of overall health status); EQ_VAS = EuroQol – Visual Analogue Scale (a measure of general health). Knee flexion = knee flexion range of motion. N represents the number of participants assigned to each trajectory cluster by the LCGA model. Due to missing data and the use of complete-case analysis in the Kruskal–Wallis and Dunn tests, estimates were derived from data from 73% to 100% of participants in the assigned trajectory cluster

#### Preoperative factors associated with recovery trajectory clusters

Each additional 1,000 preoperative steps was associated with 1.84 times higher odds of following a high-absolute recovery trajectory versus a low-absolute recovery trajectory (OR = 1.84, 95% CI 1.24 − 2.72) (Table 3). UKR, compared with TKR, was associated with higher odds of belonging to a high-absolute recovery trajectory (OR = 24.83, 95% CI 3.07–200.94) and a moderate-absolute recovery trajectory (OR = 18.17, 95% CI: 2.77–119.23) versus a low-absolute recovery trajectory.

When recovery trajectories were defined relative to preoperative step-counts rather than absolute step-counts, UKR was associated with higher odds (compared to TKR) of being in the high-relative (OR = 13.30, 95% CI 1.99–88.77) and moderate-relative recovery trajectories (OR = 8.66, 95% CI 1.28–58.64) than a low-relative recovery trajectory. There was no evidence of other preoperative factors associated with recovery trajectories (Supplementary File-7).

**Fig. 3:**
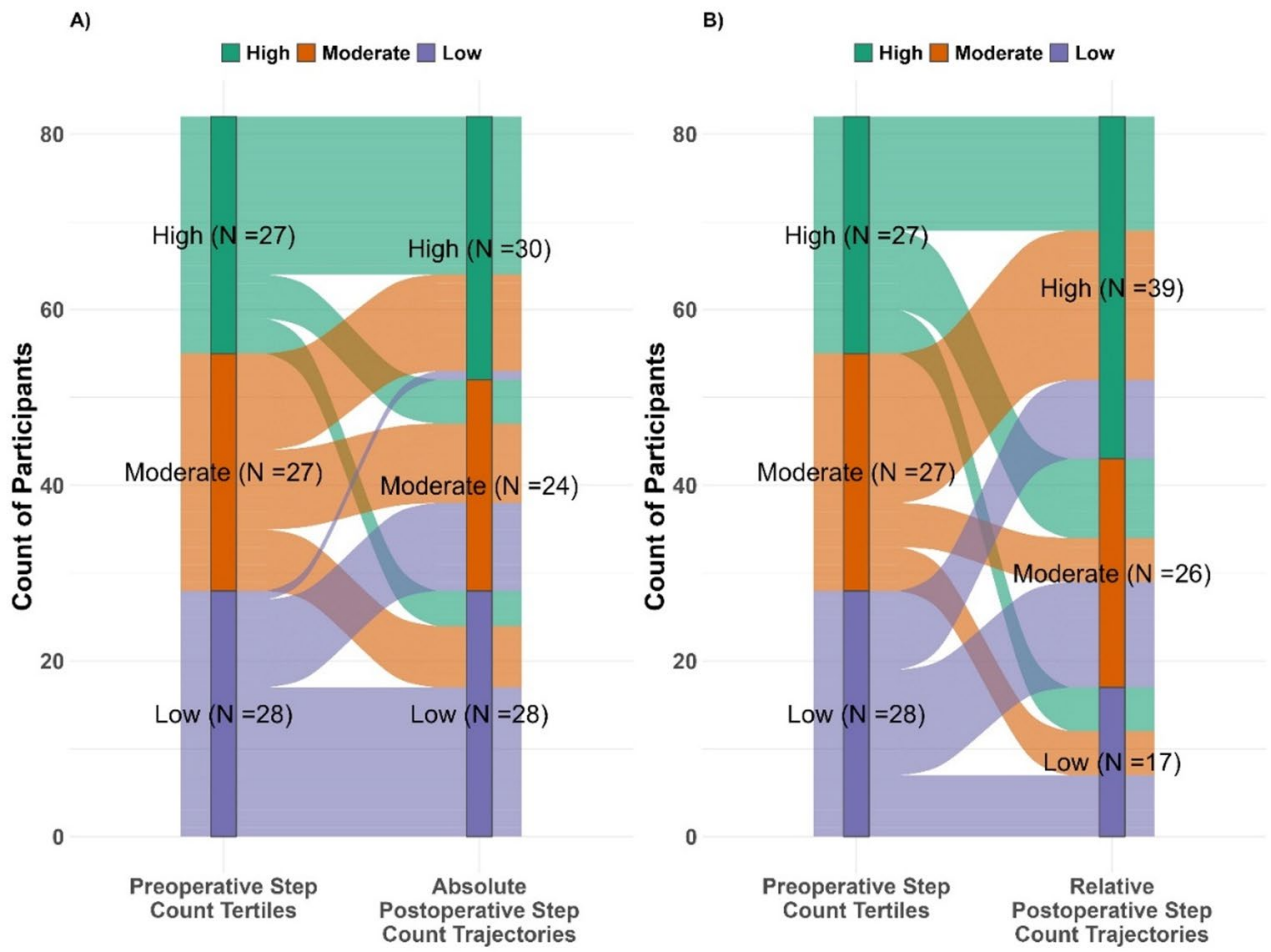
Transition of participants from preoperative step-count tertiles to postoperative recovery trajectories within six weeks after knee replacement. The left panel (**A**) shows the transition from preoperative step-count tertiles to absolute step-count recovery trajectories, and the right panel (**B**) shows the transition from preoperative step-count tertiles to relative step-count recovery trajectories

After dividing participants into preoperative step-count tertiles (median steps: high = 9,910; medium = 5,376; low = 2,558), 67% of those in the high tertile ended up in the high-absolute recovery trajectory and 48% in the high-relative recovery trajectory (Fig. 3A and B, respectively). Conversely, only 4% of those in the low tertile ended up in the high-absolute recovery trajectory while 32% were in the high-relative recovery trajectory.

**Table 3:** Association of preoperative factors with postoperative absolute step-count recovery trajectories (multivariable analysis)

Preoperative factors	High recovery (*N* = 30)		Moderate recovery (*N* = 24)	
	Odd ratio (95%CI)	*P* value	Odd ratio (95%CI)	*P* value
Age, 42–60	Reference	–	Reference	–
Age, > 60–72	2.98 (0.35–25.23)	0.316	10.81 (0.84–138.74)	0.068
Age, > 72–89	0.58 (0.06–5.98)	0.646	3.66 (0.29–46.17)	0.315
BMI, Normal	Reference	–	Reference	–
BMI, Overweight	0.18 (0.008–4.34)	0.294	0.84 (0.06–11.84)	0.897
BMI Obesity Class I to III	0.97 (0.06–15.41)	0.983	0.36 (0.03–5.00)	0.446
EQ-5D-3L index (per 0.1-unit increase)	1.11 (0.52–2.37)	0.792	1.49 (0.68–3.28)	0.318
Oxford Knee Score	1.10 (0.92–1.31)	0.292	0.95 (0.79–1.13)	0.545
EQ-VAS	0.98 (0.93–1.04)	0.597	1.01 (0.95–1.07)	0.753
Step-count (per 1000-unit increase)	1.84 (1.24–2.72)	0.002*	1.38 (0.96–2.00)	0.083
Sex, F	Reference	–	Reference	–
Sex, M	4.17 (0.66–26.35)	0.129	0.42 (0.07–2.45)	0.335
Surgery, TKR	Reference	–	Reference	–
Surgery, UKR	24.83 (3.07–200.96)	0.003*	18.17 (2.77–119.23)	0.003*

*BMI* body mass index, *CI* confidence interval, *F* Female, *M* Male, *TKR* total knee replacement, *UKR* unicompartmental knee replacement, *EQ-5D-3L index*  EuroQol 5-Dimension 3-Level United Kingdom index score (a measure of overall health status). *EQ-VAS* EuroQol visual analogue scale (a measure of general health)

Age is measured in years. The low-recovery group served as the reference group for all trajectory comparisons

*P value < 0.05. Reported associations are per 1-unit increase in the preoperative factor for continuous variables, or as noted in the results. N represents the number of participants assigned to each trajectory cluster by the LCGA model. Multinomial regression used complete-case analysis, excluding observations with missing covariate data, resulting in estimates based on 68 participants (24 high recovery, 20 moderate recovery, and 24 low recovery)

## Discussion

This secondary analysis examined step-count recovery during the first six weeks after knee replacement (UKR or TKR) in 82 patients with knee OA, using both absolute postoperative step-count and relative step-count (postoperative/preoperative). Although step-count improved in all participants compared with the first postoperative week, only 32% returned to their preoperative step-count by six weeks (38% UKR, 24% TKR). We identified three trajectories for both absolute and relative step-count, each with a similar shape through time. The high-recovery groups had rapid improvement from week one; the moderate-recovery group demonstrated slower but steady gains; and the low-recovery groups showed minimal progress until week three but with subsequent steady improvement. Patients in the high-absolute recovery trajectory had better OKS, higher EQ-5D-3L, and lower first-week pain, with the moderate-relative recovery trajectory also demonstrating better health outcomes than those in the low absolute or relative recovery trajectory. Those with higher preoperative steps were more likely to be in high-absolute recovery group, while those undergoing UKR were more likely to be in the high- or moderate-recovery clusters for both absolute and relative step trajectories. These finding indicates variation in step-count recovery during the early postoperative phase, with members of the low-recovery trajectory for both step measures at risk of slower recovery and poorer patient-reported outcomes at six weeks post-knee replacement.

Our findings suggest that step-count recovery within six weeks after knee replacement varies across individuals. All patients showed an immediate postoperative decline followed by gradual improvement, in keeping with previous studies [22–28, 47]. In our cohort, approximately one-third of participants returned to preoperative steps by week six. This was similar to a study by Twiggs et al. that found 44% of patients surpassed preoperative step-counts by week six, albeit with substantial individual variation [24]. Although some studies have described patients surpassing preoperative levels during this period [30–32], the extent of individual variation and the proportion of the population achieving this were not reported. Our study extends the existing literature by demonstrating that different recovery trajectories exist during the early postoperative period and is in line with other studies that found heterogeneity in longer-term recovery trajectories [33, 34]. Collectively, these findings show that recovery does not follow a uniform course and suggests that patients may benefit from tailored support and education based on their individual recovery pathway.

Trajectory cluster membership was strongly associated with preoperative activity level and surgery type, and patients in higher (absolute and relative) recovery trajectories reported better postoperative outcomes. We found that individuals with higher preoperative step-count and those undergoing UKR were more likely to follow high-absolute recovery trajectories, consistent with Lyman et al. (2020) [33], who found that patients with higher preoperative step-count were more likely to belong to a high-absolute recovery trajectory than a low one, and with Kugelman et al. [48], who reported faster gait recovery after UKR than TKR. Stratifying preoperative step-count into tertiles and mapping them to absolute and relative step-count postoperative trajectories revealed not only that individuals in the lowest tertile – as expected – were unlikely to achieve a high-absolute recovery trajectory, but also that they were less likely to achieve a high-relative trajectory compared to those in the higher preoperative step-count tertiles. This suggests that individuals who were more active before surgery tend to recover more rapidly afterwards, potentially due to factors such as general health, comorbidities and frailty. This information might support prehabilitation and promoting an active lifestyle before surgery, while also providing realistic expectations of postoperative recovery based on baseline activity levels.

This study has several limitations. The sample size was modest (82 participants), which led to poor precision and wide confidence intervals for the multivariable analysis. Although the study was conducted at a single site, which may limit external validity, this design reduces variability in surgical technique, postoperative care, and likely outcomes. The study period (February 2020–July 2021) overlapped with the COVID-19 pandemic, during which movement restrictions [49] likely influenced activity levels. The relative short study duration of six weeks and our exploration of within-person change is likely to minimise the impact of government policy on mobility due to the pandemic, although there may still be a residual effect. The absence of information on the month or season of data collection limited our ability to explore potential weather-related variation in step-counts. Additionally, data on other potentially influential factors such as medication use, physiotherapy intensity, and psychosocial factors (e.g., anxiety related to wound care or mobility) were not captured and could not be evaluated. The step-count algorithm used was validated for measuring steps in healthy adults [50] and not in a population with knee OA or post knee replacement. These populations are prone to altered gait patterns [51], making their gait different from that of healthy adults. Similar accelerometry devices have, however, shown adequate construct validity for step-count in people with knee OA [52]. Furthermore, it is possible that the use of walking aids in the perioperative may change arm swing, which could lead to an underestimate of true step-count. We would anticipate that this would impact our participants earlier in their recovery, with the effect diminishing as their recovery improves. However, this potential underestimation is unlikely to affect trajectory assignment or distinction, which are based on patterns over time [53]. As this was a secondary analysis, information on the exact timing of preoperative step-count collection relative to surgery was not available, preventing comparison of activity levels closer to the time of surgery. Despite these, recorded step-counts were within the range reported in previous postoperative recovery studies [24–28, 33], therefore raising no major concerns for non-representativeness. There is a risk that the study participants were more active (e.g., younger and with lower BMI), which could lead to underrepresentation of individuals who do not recover at the same level as those with a more active lifestyle. Nonetheless, despite these limitations, daily monitoring for six weeks per participant provided more than 3,000 daily observations, enabling a detailed characterisation of early postoperative recovery trajectories, examination of the influence of preoperative factors on trajectory membership, and illustration of how preoperative activity tertiles relate to postoperative trajectory membership. Our study contributes to the limited evidence on early postoperative activity recovery and offers insights that may help inform clinical decision-making prior to and during this critical early postoperative period.

Although we identified three recovery trajectories, recovery likely exists along a spectrum and should be interpreted not as wholly distinct trajectories but as three broadly representative patterns along a continuum. The latent class growth modelling approach used in this study assigns individuals to groups based on the probability of membership, though we know that some individuals may fall somewhere between the identified trajectories. Our finding that UKR surgery type is associated with a more favourable recovery trajectory should not be interpreted as a causal factor for trajectory assignment, as this relationship may be confounded by indication (where the reason for choosing UKR versus TKR (e.g., extent of knee OA) is itself associated with the outcome). Nevertheless, the trajectories illustrate that recovery can vary, with some patients showing improvement soon after surgery while others experience delays before achieving measurable gains. The extent and speed of response, and the proportions of patients that follow each trajectory, are now estimated from this study, allowing clinicians to explain expected recovery to support informed decision making. These insights can be helpful when educating people before a knee replacement surgery and when monitoring their recovery during the first six weeks. None of the identified trajectories appeared to have fully plateaued by week six, indicating that recovery continues beyond this time point. This underscores the need for longer follow-up to characterise longer-term recovery patterns and trajectories. Activity data from consumer accelerometry, when linked to verified surgical data, could support such work at scale, particularly as these devices are increasingly used to measure PA in the general population.

In conclusion, one in three individuals regained their preoperative step-counts by six weeks after knee replacement. Those undergoing UKR and those with higher preoperative step-counts were more likely to have a high- or moderate-absolute recovery trajectory, characterised by faster and greater improvements than the low-absolute recovery trajectory. Participants in the high- and moderate-recovery trajectories reported less pain, better OKS scores, and better health status than those in the low-recovery cluster. Although the results are based on a single site and a small sample size, thereby warranting caution in interpretation and replication at a larger scale, these findings could help clinicians set expectations. This can allow patients to make more informed decisions and shape future work on early identification of treatment response to support targeted interventions.

## Electronic Supplementary Material

Below is the link to the electronic supplementary material.

Supplementary Material 1

Supplementary Material 2

Supplementary Material 3

Supplementary Material 4

Supplementary Material 5

Supplementary Material 6

Supplementary Material 7

Supplementary Material 8

## Data Availability

The data supporting the findings of this study are not openly available due to their sensitivity but are available from the authors upon reasonable request and with permission from the Nuffield Department of Orthopaedics, Rheumatology and Musculoskeletal Sciences, University of Oxford data governance team.

## Materials and/or code availability

The code used in this study is available upon request from the corresponding author.
