# Supplementary material for: Step count recovery patterns in the first six weeks after knee replacement in individuals with knee osteoarthritis: a secondary analysis of a prospective observational cohort study using wrist-worn accelerometry
Source: Rheumatol Int. 2026 Jun 4;46(6):131. doi: 10.1007/s00296-026-06135-y (PMC13233972; doi:10.1007/s00296-026-06135-y)
Supplement: Supplementary file 1 — Supplementary Material 1 [file 296_2026_6135_MOESM1_ESM.docx]

**Supplementary File 1:** Details of step count derivation

**Article Title*:*** Step count recovery patterns in the first six weeks after knee replacement in individuals with knee osteoarthritis: a secondary analysis of a prospective observational cohort study using wrist-worn accelerometry

**Journal Name:** Rheumatology International

**Author Information**

Ayobami E. Olanrewaju, ayobami.olanrewaju@postgrad.manchester.ac.uk, 0000-0002-4520-7019^1,2^; Emma Pritchard, emma.pritchard@manchester.ac.uk, 0000-0002-0963-9260^1^; Shuai Shao, shuai.shao@manchester.ac.uk, 0009-0002-7028-0944^1^; Andrew J. Price, andrew.price@ndorms.ox.ac.uk, 0000-0002-4258-5866^3^; Aiden Doherty, aiden.doherty@ndph.ox.ac.uk, 0000-0003-1840-0451^4^; Sabine N. van der Veer, sabine.vanderveer@manchester.ac.uk, 0000-0003-0929-436X^1^; David C. Wong, d.c.wong@leeds.ac.uk, 0000-0001-8117-9193^5^; Scott R. Small, scott.small@ndorms.ox.ac.uk, 0000-0003-3603-8062^3,4^; Stephanie R. Filbay, stephanie.filbay@unimelb.edu.au, 0000-0002-9624-0791^2^; William G. Dixon, will.dixon@manchester.ac.uk, 0000-0001-5881-4857^1,6^

1. University of Manchester, School of Health Sciences, Division of Informatics, Imaging and Data Sciences, M13 9PT, Manchester, United Kingdom.
2. University of Melbourne, Centre for Health, Exercise and Sports Medicine, Department of Physiotherapy, Parkville, Victoria 3000, Melbourne, Australia.
3. University of Oxford, Nuffield Department of Orthopaedics, Rheumatology and Musculoskeletal Sciences, Oxford, United Kingdom.
4. University of Oxford, Nuffield Department of Population Health, Oxford, United Kingdom.
5. University of Leeds, Leeds Institute of Health Sciences, Leeds, United Kingdom.
6. NIHR Manchester Biomedical Research Centre, Manchester University NHS Foundation Trust, Manchester Academic Health Science Centre.

**Corresponding Author**

Ayobami E. Olanrewaju,

Division of Informatics, Imaging and Data Sciences, School of Health Sciences, University of Manchester, M13 9GB, Manchester, United Kingdom.

Email: ayobami.olanrewaju@postgrad.manchester.ac.uk.

Data processing was conducted in three steps to derive physical activity metrics. First, acceleration was calibrated to gravity [1] and machine noise was removed using a 20 Hz Butterworth low-pass filter. Second, non-wear time, defined as > 60 minutes with zero vector magnitude, was excluded. Vector magnitude represents overall acceleration, computed as the Euclidean norm of the three accelerometer axes. Third, physical activity metrics were estimated using established methods [2], including the UK Biobank analysis tool [3], a validated open-source step detection algorithm [4], and machine-learning approaches [5]. For this study, daily step-count was selected as the primary physical activity measure.

**References**

1. van Hees VT, Fang Z, Langford J, Assah F, Mohammad A, da Silva ICM, et al. Autocalibration of accelerometer data for free-living physical activity assessment using local gravity and temperature: an evaluation on four continents. J Appl Physiol (1985). 2014;117:738–44. https://doi.org/10.1152/japplphysiol.00421.2014

2. Doherty A, Jackson D, Hammerla N, Plötz T, Olivier P, Granat MH, et al. Large Scale Population Assessment of Physical Activity Using Wrist Worn Accelerometers: The UK Biobank Study. PLoS One. 2017;12:e0169649. https://doi.org/10.1371/journal.pone.0169649

3. Doherty A, Chan S, Yuan H, Walmsley R. accelerometer: A Python Toolkit for Extracting Physical Activity and Behavior Metrics from Wearable Sensor Data [Internet]. 2020 [cited 2025 Jan 16]. https://doi.org/10.5281/zenodo.14515076

4. Small SR, Chan S, Walmsley R, von Fritsch L, Acquah A, Mertes G, et al. Self-Supervised Machine Learning to Characterise Step Counts from Wrist-Worn Accelerometers in the UK Biobank. Medicine & Science in Sports & Exercise. 2023;10.1249/MSS.0000000000003478. https://doi.org/10.1249/MSS.0000000000003478

5. Walmsley R, Chan S, Smith-Byrne K, Ramakrishnan R, Woodward M, Rahimi K, et al. Reallocation of time between device-measured movement behaviours and risk of incident cardiovascular disease. Br J Sports Med. BMJ Publishing Group Ltd and British Association of Sport and Exercise Medicine; 2022;56:1008–17. https://doi.org/10.1136/bjsports-2021-104050
