# Supplementary material for: Step count recovery patterns in the first six weeks after knee replacement in individuals with knee osteoarthritis: a secondary analysis of a prospective observational cohort study using wrist-worn accelerometry
Source: Rheumatol Int. 2026 Jun 4;46(6):131. doi: 10.1007/s00296-026-06135-y (PMC13233972; doi:10.1007/s00296-026-06135-y)
Supplement: Supplementary file 4 — Supplementary Material 4 [file 296_2026_6135_MOESM4_ESM.docx]

**Supplementary File 4:** Proportion of people exceeding their preoperative step-count by age and sex category

**Article Title*:*** Step count recovery patterns in the first six weeks after knee replacement in individuals with knee osteoarthritis: a secondary analysis of a prospective observational cohort study using wrist-worn accelerometry

**Journal Name:** Rheumatology International

**Author Information**

Ayobami E. Olanrewaju, ayobami.olanrewaju@postgrad.manchester.ac.uk, 0000-0002-4520-7019^1,2^; Emma Pritchard, emma.pritchard@manchester.ac.uk, 0000-0002-0963-9260^1^; Shuai Shao, shuai.shao@manchester.ac.uk, 0009-0002-7028-0944^1^; Andrew J. Price, andrew.price@ndorms.ox.ac.uk, 0000-0002-4258-5866^3^; Aiden Doherty, aiden.doherty@ndph.ox.ac.uk, 0000-0003-1840-0451^4^; Sabine N. van der Veer, sabine.vanderveer@manchester.ac.uk, 0000-0003-0929-436X^1^; David C. Wong, d.c.wong@leeds.ac.uk, 0000-0001-8117-9193^5^; Scott R. Small, scott.small@ndorms.ox.ac.uk, 0000-0003-3603-8062^3,4^; Stephanie R. Filbay, stephanie.filbay@unimelb.edu.au, 0000-0002-9624-0791^2^; William G. Dixon, will.dixon@manchester.ac.uk, 0000-0001-5881-4857^1,6^

1. University of Manchester, School of Health Sciences, Division of Informatics, Imaging and Data Sciences, M13 9PT, Manchester, United Kingdom.
2. University of Melbourne, Centre for Health, Exercise and Sports Medicine, Department of Physiotherapy, Parkville, Victoria 3000, Melbourne, Australia.
3. University of Oxford, Nuffield Department of Orthopaedics, Rheumatology and Musculoskeletal Sciences, Oxford, United Kingdom.
4. University of Oxford, Nuffield Department of Population Health, Oxford, United Kingdom.
5. University of Leeds, Leeds Institute of Health Sciences, Leeds, United Kingdom.
6. NIHR Manchester Biomedical Research Centre, Manchester University NHS Foundation Trust, Manchester Academic Health Science Centre.

**Corresponding Author**

Ayobami E. Olanrewaju,

Division of Informatics, Imaging and Data Sciences, School of Health Sciences, University of Manchester, M13 9GB, Manchester, United Kingdom.

Email: ayobami.olanrewaju@postgrad.manchester.ac.uk.


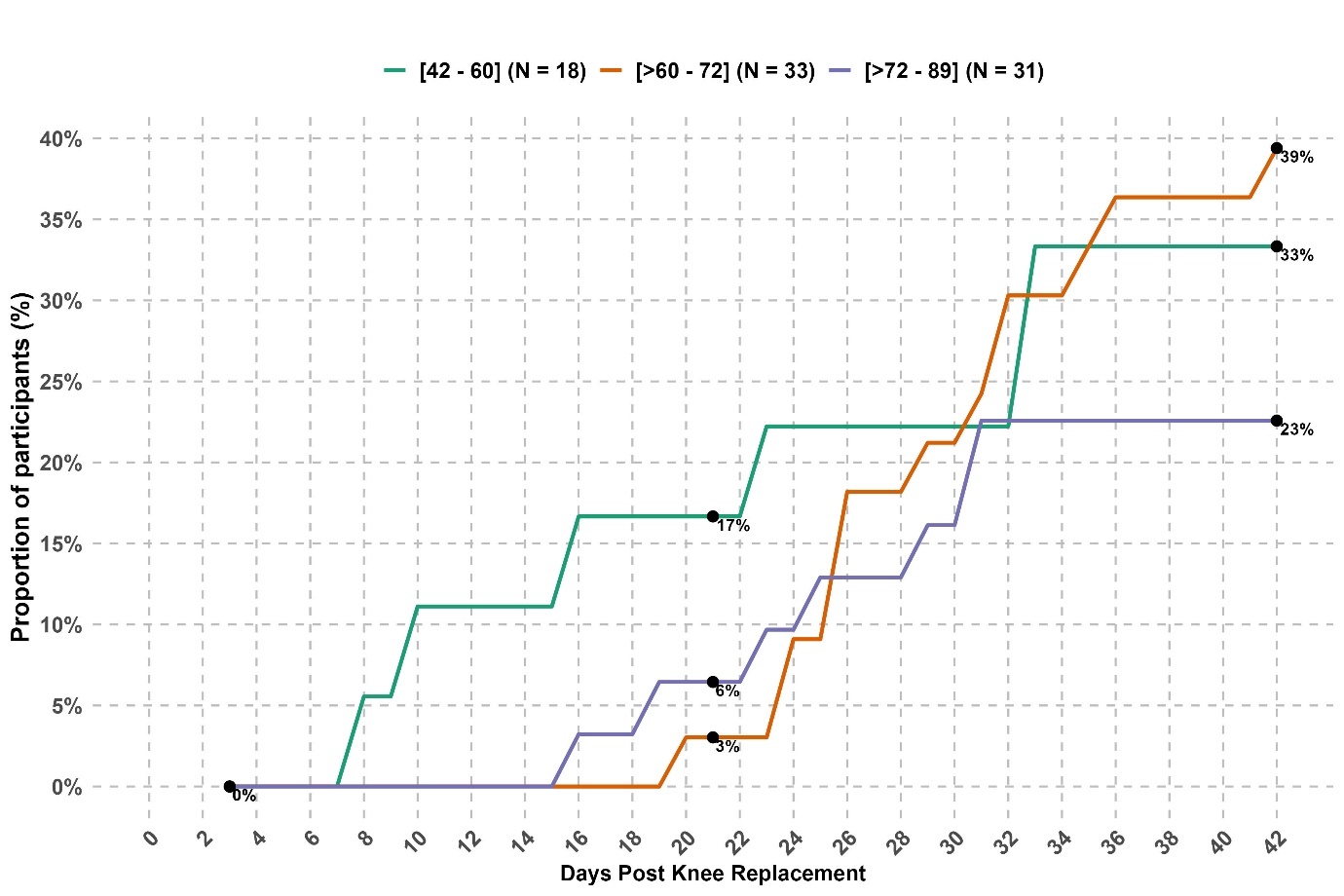


**Fig. 1:** Proportion of participants exceeding their preoperative step-count at least once during the six weeks postoperative period, by age category.


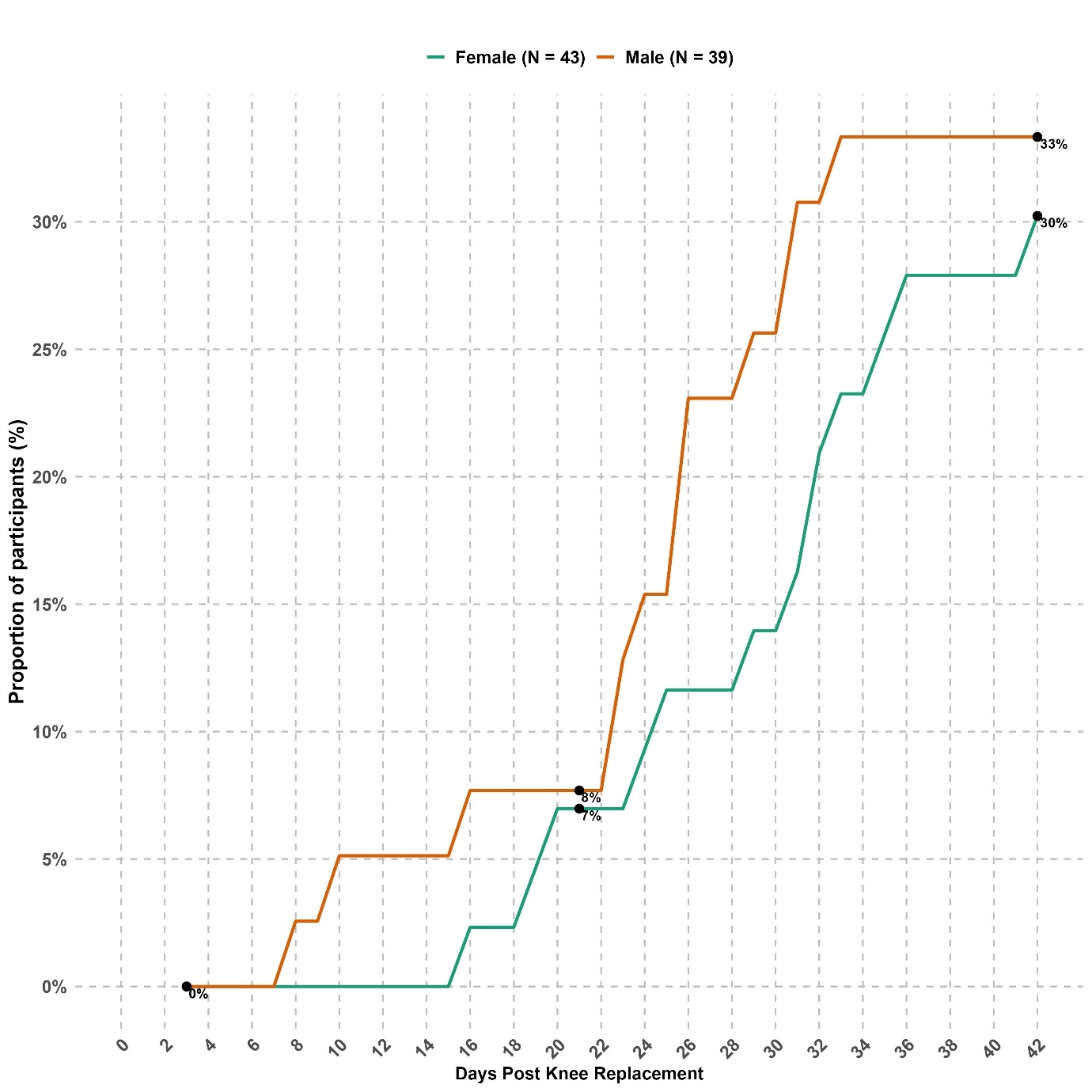


**Fig. 2:** Proportion of participants exceeding their preoperative step-count at least once during the six weeks postoperative period, by sex category.
