# Supplementary material for: Step count recovery patterns in the first six weeks after knee replacement in individuals with knee osteoarthritis: a secondary analysis of a prospective observational cohort study using wrist-worn accelerometry
Source: Rheumatol Int. 2026 Jun 4;46(6):131. doi: 10.1007/s00296-026-06135-y (PMC13233972; doi:10.1007/s00296-026-06135-y)
Supplement: Supplementary file 5 — Supplementary Material 5 [file 296_2026_6135_MOESM5_ESM.docx]

**Supplementary File 5:** Model fit parameters for absolute step count recovery trajectory of two to five clusters

**Article Title*:*** Step count recovery patterns in the first six weeks after knee replacement in individuals with knee osteoarthritis: a secondary analysis of a prospective observational cohort study using wrist-worn accelerometry

**Journal Name:** Rheumatology International

**Author Information**

Ayobami E. Olanrewaju, ayobami.olanrewaju@postgrad.manchester.ac.uk, 0000-0002-4520-7019^1,2^; Emma Pritchard, emma.pritchard@manchester.ac.uk, 0000-0002-0963-9260^1^; Shuai Shao, shuai.shao@manchester.ac.uk, 0009-0002-7028-0944^1^; Andrew J. Price, andrew.price@ndorms.ox.ac.uk, 0000-0002-4258-5866^3^; Aiden Doherty, aiden.doherty@ndph.ox.ac.uk, 0000-0003-1840-0451^4^; Sabine N. van der Veer, sabine.vanderveer@manchester.ac.uk, 0000-0003-0929-436X^1^; David C. Wong, d.c.wong@leeds.ac.uk, 0000-0001-8117-9193^5^; Scott R. Small, scott.small@ndorms.ox.ac.uk, 0000-0003-3603-8062^3,4^; Stephanie R. Filbay, stephanie.filbay@unimelb.edu.au, 0000-0002-9624-0791^2^; William G. Dixon, will.dixon@manchester.ac.uk, 0000-0001-5881-4857^1,6^

1. University of Manchester, School of Health Sciences, Division of Informatics, Imaging and Data Sciences, M13 9PT, Manchester, United Kingdom.
2. University of Melbourne, Centre for Health, Exercise and Sports Medicine, Department of Physiotherapy, Parkville, Victoria 3000, Melbourne, Australia.
3. University of Oxford, Nuffield Department of Orthopaedics, Rheumatology and Musculoskeletal Sciences, Oxford, United Kingdom.
4. University of Oxford, Nuffield Department of Population Health, Oxford, United Kingdom.
5. University of Leeds, Leeds Institute of Health Sciences, Leeds, United Kingdom.
6. NIHR Manchester Biomedical Research Centre, Manchester University NHS Foundation Trust, Manchester Academic Health Science Centre.

**Corresponding Author**

Ayobami E. Olanrewaju,

Division of Informatics, Imaging and Data Sciences, School of Health Sciences, University of Manchester, M13 9GB, Manchester, United Kingdom.

Email: ayobami.olanrewaju@postgrad.manchester.ac.uk.

**Table 1:** Model fit parameters for the absolute step count trajectory models.

| C | LogLik | BIC | Entropy | %C1 (PP) | %C2 (PP) | %C3 (PP) | %C4 (PP) | *%C5 (PP) |
| --- | --- | --- | --- | --- | --- | --- | --- | --- |
| 2 | -26170.34 | 52397.96 | 0.987 | 51.22(1.000) | 48.78(0.993) | - | - | - |
| 3 | -25809.17 | 51693.25 | 0.983 | 36.59(0.987) | 34.15(0.994) | 29.27(0.999) | - | - |
| 4 | -25526.67 | 51145.87 | 0.989 | 19.51(0.988) | 35.37(0.998) | 30.49(0.996) | 14.63(0.998) | - |
| 5 | -25568.28 | 51246.72 | 0.994 | 32.93(0.999) | 29.27(0.998) | 26.83(0.992) | 10.98(1.000) | - |

LogLik = log-likelihood; measures how well the model fits the observed data. C = number of classes (i.e. groups) used when fitting the model. BIC = Bayesian Information Criterion; a metric for comparing statistical models fitted to the same dataset. Entropy = measures how well a model classifies individuals into classes. PP = posterior class-membership probabilities; the probability that an individual belongs to a given trajectory class. *The five-class model identified only four trajectory classes.
