# Supplementary material for: Step count recovery patterns in the first six weeks after knee replacement in individuals with knee osteoarthritis: a secondary analysis of a prospective observational cohort study using wrist-worn accelerometry
Source: Rheumatol Int. 2026 Jun 4;46(6):131. doi: 10.1007/s00296-026-06135-y (PMC13233972; doi:10.1007/s00296-026-06135-y)
Supplement: Supplementary file 6 — Supplementary Material 6 [file 296_2026_6135_MOESM6_ESM.docx]

**Supplementary File 6:** Association between postoperative outcomes and relative step count recovery trajectories

**Article Title*:*** Step count recovery patterns in the first six weeks after knee replacement in individuals with knee osteoarthritis: a secondary analysis of a prospective observational cohort study using wrist-worn accelerometry

**Journal Name:** Rheumatology International

**Author Information**

Ayobami E. Olanrewaju, ayobami.olanrewaju@postgrad.manchester.ac.uk, 0000-0002-4520-7019^1,2^; Emma Pritchard, emma.pritchard@manchester.ac.uk, 0000-0002-0963-9260^1^; Shuai Shao, shuai.shao@manchester.ac.uk, 0009-0002-7028-0944^1^; Andrew J. Price, andrew.price@ndorms.ox.ac.uk, 0000-0002-4258-5866^3^; Aiden Doherty, aiden.doherty@ndph.ox.ac.uk, 0000-0003-1840-0451^4^; Sabine N. van der Veer, sabine.vanderveer@manchester.ac.uk, 0000-0003-0929-436X^1^; David C. Wong, d.c.wong@leeds.ac.uk, 0000-0001-8117-9193^5^; Scott R. Small, scott.small@ndorms.ox.ac.uk, 0000-0003-3603-8062^3,4^; Stephanie R. Filbay, stephanie.filbay@unimelb.edu.au, 0000-0002-9624-0791^2^; William G. Dixon, will.dixon@manchester.ac.uk, 0000-0001-5881-4857^1,6^

1. University of Manchester, School of Health Sciences, Division of Informatics, Imaging and Data Sciences, M13 9PT, Manchester, United Kingdom.
2. University of Melbourne, Centre for Health, Exercise and Sports Medicine, Department of Physiotherapy, Parkville, Victoria 3000, Melbourne, Australia.
3. University of Oxford, Nuffield Department of Orthopaedics, Rheumatology and Musculoskeletal Sciences, Oxford, United Kingdom.
4. University of Oxford, Nuffield Department of Population Health, Oxford, United Kingdom.
5. University of Leeds, Leeds Institute of Health Sciences, Leeds, United Kingdom.
6. NIHR Manchester Biomedical Research Centre, Manchester University NHS Foundation Trust, Manchester Academic Health Science Centre.

**Corresponding Author**

Ayobami E. Olanrewaju,

Division of Informatics, Imaging and Data Sciences, School of Health Sciences, University of Manchester, M13 9GB, Manchester, United Kingdom.

Email: ayobami.olanrewaju@postgrad.manchester.ac.uk.

**Table 1:** Postoperative patient-reported outcomes by relative step count trajectory

| Postoperative Outcomes | High Relative Recovery  (N = 39) | Moderate Relative Recovery  (N = 26) | Low Relative Recovery  (N = 17) | ^*^Kruskal Wallis | | *Dunn’s Test* | | |
| --- | --- | --- | --- | --- | --- | --- | --- | --- |
|  |  |  |  | KW  $\chi^{2}$ | *P* value | High Relative Recovery Vs Moderate (*P* value) | High Relative Recovery Vs Low (*P* value) | Moderate Relative Recovery Vs Low (*P* value) |
| Maximum pain (week one) | 7.0 (4.0) | 8.0 (2.0) | 7.5 (2.2) | 0.38 | 0.826 | - | - | - |
| Median pain  (week one) | 6.0 (3.8) | 6.0 (3.2) | 7.0 (2.2) | 2.43 | 0.297 | - | - | - |
| Maximum pain (week six) | 2.0 (2.5) | 4.0 (2.5) | 4.0 (1.2) | 6.55 | 0.038 | 0.143 | 0.061 | 0.542 |
| Median pain  (week six) | 2.0 (2.0) | 3.0 (2.0) | 3.0 (1.2) | 4.95 | 0.084 | - | - | - |
| Oxford Knee Score | 33.0 (6.8) | 29.5 (9.7) | 23.0 (6.4) | 16.85 | <0.001 | 0.511 | <0.001 | 0.003 |
| EQ_VAS | 80.0 (22.6) | 81.0 (15) | 60.0 (31.3) | 12.51 | 0.002 | 0.259 | 0.012 | 0.001 |
| EQ_5D_3L index | 0.7 (0.1) | 0.7 (0.1) | 0.6 (0.2) | 13.38 | 0.001 | 0.167 | <0.001 | 0.043 |
| Knee flexion | 75.0 (11.7) | 80.0 (10.0) | 60.0 (36) | 2.52 | 0.284 | - | - | - |

All outcomes were reported as median (interquartile range). KW = Kruskal–Wallis test, a nonparametric test for group differences. Dunn’s test = post-hoc test used to determine where differences exist between the identified trajectories. *Degree of Freedom for the Kruskal Wallis test = 2. $\chi^{2}$ = chi-square. High = High recovery trajectory; Moderate = moderate recovery trajectory; Low = Low recovery trajectory; EQ_5D_3L index = EuroQol 5-Dimension 3-Level United Kingdom index score (a measure of overall health status); EQ_VAS = EuroQol – Visual Analogue Scale (a measure of general health). Knee flexion = knee flexion range of motion. N represents the number of participants assigned to each trajectory cluster by the LCGA model. Due to missing data and the use of complete-case analysis in the Kruskal–Wallis and Dunn tests, estimates were derived from data from 58% to 97% of participants in the assigned trajectory cluster.
