# Supplementary material for: Step count recovery patterns in the first six weeks after knee replacement in individuals with knee osteoarthritis: a secondary analysis of a prospective observational cohort study using wrist-worn accelerometry
Source: Rheumatol Int. 2026 Jun 4;46(6):131. doi: 10.1007/s00296-026-06135-y (PMC13233972; doi:10.1007/s00296-026-06135-y)
Supplement: Supplementary file 7 — Supplementary Material 7 [file 296_2026_6135_MOESM7_ESM.docx]

**Supplementary File 7:** Preoperative factors associated with relative step count recovery trajectories

**Article Title*:*** Step count recovery patterns in the first six weeks after knee replacement in individuals with knee osteoarthritis: a secondary analysis of a prospective observational cohort study using wrist-worn accelerometry

**Journal Name:** Rheumatology International

**Author Information**

Ayobami E. Olanrewaju, ayobami.olanrewaju@postgrad.manchester.ac.uk, 0000-0002-4520-7019^1,2^; Emma Pritchard, emma.pritchard@manchester.ac.uk, 0000-0002-0963-9260^1^; Shuai Shao, shuai.shao@manchester.ac.uk, 0009-0002-7028-0944^1^; Andrew J. Price, andrew.price@ndorms.ox.ac.uk, 0000-0002-4258-5866^3^; Aiden Doherty, aiden.doherty@ndph.ox.ac.uk, 0000-0003-1840-0451^4^; Sabine N. van der Veer, sabine.vanderveer@manchester.ac.uk, 0000-0003-0929-436X^1^; David C. Wong, d.c.wong@leeds.ac.uk, 0000-0001-8117-9193^5^; Scott R. Small, scott.small@ndorms.ox.ac.uk, 0000-0003-3603-8062^3,4^; Stephanie R. Filbay, stephanie.filbay@unimelb.edu.au, 0000-0002-9624-0791^2^; William G. Dixon, will.dixon@manchester.ac.uk, 0000-0001-5881-4857^1,6^

1. University of Manchester, School of Health Sciences, Division of Informatics, Imaging and Data Sciences, M13 9PT, Manchester, United Kingdom.
2. University of Melbourne, Centre for Health, Exercise and Sports Medicine, Department of Physiotherapy, Parkville, Victoria 3000, Melbourne, Australia.
3. University of Oxford, Nuffield Department of Orthopaedics, Rheumatology and Musculoskeletal Sciences, Oxford, United Kingdom.
4. University of Oxford, Nuffield Department of Population Health, Oxford, United Kingdom.
5. University of Leeds, Leeds Institute of Health Sciences, Leeds, United Kingdom.
6. NIHR Manchester Biomedical Research Centre, Manchester University NHS Foundation Trust, Manchester Academic Health Science Centre.

**Corresponding Author**

Ayobami E. Olanrewaju,

Division of Informatics, Imaging and Data Sciences, School of Health Sciences, University of Manchester, M13 9GB, Manchester, United Kingdom.

Email: ayobami.olanrewaju@postgrad.manchester.ac.uk.

**Table 1:** Association of preoperative factors with relative step count recovery trajectories (multivariable analysis)

|  | High Recovery (N=39) | | Moderate Recovery (N= 26) | |
| --- | --- | --- | --- | --- |
| Preoperative Factors | **Odd ratio(95%CI)** | ***P* value** | **Odd ratio(95%CI)** | ***P* value** |
| Age, 42 - 60 | Reference | - | Reference | - |
| Age, >60 - 72 | 3.24 (0.42 – 24.74) | 0.257 | 0.84 (0.09 – 7.79) | 0.881 |
| Age, >72 - 89 | 1.27 (0.16 – 9.91) | 0.823 | 0.93 (0.12 – 7.37) | 0.943 |
| BMI, Normal | Reference | - | Reference | - |
| BMI Overweight | 6.69 (0.44 – 102.55) | 0.172 | 13.57 (0.90 – 204.13) | 0.059 |
| BMI Obesity Class I to III | 2.08 (0.27 – 16.25) | 0.485 | 1.34 (0.16 – 11.53) | 0.789 |
| EQ-5D 3L index (per 0.1-unit increase) | 1.49 (0.75 – 2.96) | 0.256 | 1.26 (0.60 – 2.67) | 0.538 |
| Oxford Knee Score | 1.09 (0.92 - 1.29) | 0.318 | 1.08 (0.90 - 1.30) | 0.389 |
| EQ-VAS | 0.99 (0.93 – 1.04) | 0.668 | 1.02 (0.97 - 1.09) | 0.423 |
| Sex, F | Reference | - | Reference | - |
| Sex, M | 1.22 (0.25 – 6.07) | 0.806 | 0.77 (0.15 – 4.06) | 0.759 |
| Surgery, TKR | Reference | - | Reference | - |
| Surgery, UKR | 13.30 (1.99 – 88.77) | 0.008* | 8.66 (1.28 - 58.64) | 0.027* |

BMI: Body Mass Index; CI: confidence interval; F: Female; M: Male; TKR: Total Knee Replacement; UKR: Unicompartmental Knee Replacement. EQ-5D-3L index = EuroQol 5-Dimension 3-Level United Kingdom index score (a measure of overall health status); EQ-VAS: EuroQol Visual Analogue Scale (a measure of general health); Age is measured in years. The low-recovery group served as the reference group for all trajectory comparisons. **P* value < 0.05. Reported associations are per 1-unit increase in the preoperative factor for continuous variables, or as noted in the results. N represents the number of participants assigned to each trajectory cluster by the LCGA model. Multinomial regression used complete-case analysis, excluding observations with missing covariate data, resulting in estimates based on 68 participants (30 high recovery, 23 moderate recovery, and 15 low recovery).
